# Evaluating the impact of a novel restricted reimbursement policy for quinolone antibiotics: A time series analysis

**DOI:** 10.1186/1472-6963-12-290

**Published:** 2012-08-30

**Authors:** Braden Manns, Kevin Laupland, Marcello Tonelli, Song Gao, Brenda Hemmelgarn

**Affiliations:** 1 Departments of Medicine and Community Health Sciences, Calgary, Alberta, Canada; 2 The Libin Cardiovascular Institute of Alberta, Calgary, Alberta, Canada; 3 Alberta Kidney Disease Network, University of Calgary, Calgary, Alberta, Canada; 4 Department of Medicine, Division of Nephrology, Alberta, Canada; 5 University of Alberta, Edmonton, Alberta, Canada

**Keywords:** Formulary restriction, Antibiotic, Prior authorization, Prescription drugs

## Abstract

**Background:**

Publicly-funded drug plans often use prior authorization policies to limit drug prescribing. To guide physician prescribing of a class of antibiotics with broad antimicrobial activity (quinolone antibiotics) in accordance with new prescribing guidelines, Alberta’s provincial health ministry implemented a new mechanism for formulary restriction entitled the optional special authorization (OSA) program. We conducted an observational study to determine the impact of this new formulary restriction policy on antimicrobial prescription rates as well as any clinical consequences.

**Methods:**

Quinolone antibiotic use, and adherence with quinolone prescribing guidelines, was assessed before and after implementation of the OSA program in patients with common outpatient infections using an administrative data cohort and a chart review cohort, respectively. At the same time this policy was implemented to limit quinolone prescribing, two new quinolone antibiotics were added to the formulary. Using administrative data, we analysed a total of 397,534 unique index visits with regard to overall antibiotic utilization, and through chart review, we analysed 1681 charts of patients with infections of interest to determine the indications for quinolone usage.

**Results:**

Using segmented regression models adjusting for age, sex and physician enrollment in the OSA program, there was no statistically significant change in the monthly rate of all quinolone use (−3.5 (95% CI −5.5, 1.4) prescriptions per 1000 index visits) following implementation of the OSA program (p = 0.74). There was a significant level change in the rate of quinolone antibiotic use for urinary tract infection (−33.6 (95% CI: -23.8, -43.4) prescriptions and upper respiratory tract infection (−16.1 (95%CI: -11.6, -20.6) prescriptions per 1000 index visits. Among quinolone prescriptions identified on chart review, 42.5% and 58.5% were consistent with formulary guidelines before and after the implementation of the OSA program, respectively (p = 0.002). There was no change in hospitalization, mortality or use of physician services after implementation of the OSA program.

**Conclusions:**

Despite the addition of two new quinolone antibiotics to the formulary, we found that there was no change in the use of quinolones after implementation of a new formulary restriction policy for outpatients with common outpatient infections.

## Background

Spending for prescription drugs represents nearly 17% of Canada’s health care expenditures and is the fastest-growing component of the health care budget [1], escalating at 8.3% per year. In an attempt to guide appropriate prescribing, maximize health benefits, and limit costs, most publicly funded health care systems have developed formularies or implemented policies to optimize prescription drug use. To facilitate prescribing restrictions, formularies often use a prior authorization process (where doctors apply and receive prior approval for use of restricted medications). Traditional prior authorization programs have been shown to reduce prescription rates of several classes of medications including COX II inhibitors [2,3], respiratory drugs [4,5] and antidepressant agents [6]. However, they have been criticized as being intrusive [7], time-consuming, expensive to administer, and their effects may be temporary [8].

Increases in drug expenditures have been particularly pronounced for antimicrobial agents, and concerns also exist that their misuse and overuse may lead to antimicrobial resistance [9-11]. To limit excessive use of antibiotics, some hospitals have experimented with use of traditional prior authorization, either through a computer-assisted ordering process or prior authorization with the infectious diseases consultant on-call [12-14]. Interventions to limit outpatient prescribing of antibiotics, where the majority of use occurs, have received less attention.

A provincial strategy to improve outpatient antibiotic prescribing and reduce antimicrobial resistance was recently implemented in Alberta, Canada, and specifically recommended quinolone antibiotics (a class of oral antibiotics with broad antimicrobial activity), particularly second and third generation agents, as second line treatment for patients with community acquired pneumonia, acute exacerbation of chronic obstructive pulmonary disease (COPD), and acute sinusitis. Acknowledging that traditional prior authorization was likely infeasible, a new formulary restriction was developed (termed “optional special authorization” (OSA)) in conjunction with changes to prescribing restrictions for quinolone antibiotics. We conducted an observational study to determine the impact of this new formulary restriction policy on antimicrobial prescription rates as well as any clinical and economic consequences.

## Methods

### Study objectives

Our primary objective was to measure the impact of a new reimbursement policy aimed at restricting the use of quinolones to defined subgroups of patients with common outpatient infections including urinary tract infection (UTI), upper respiratory tract infection (URTI), acute exacerbation of chronic bronchitis (AECB) and community acquired pneumonia. The secondary objective was to compare the proportion of quinolone prescriptions which were consistent with the restricted reimbursement prescribing criteria, before and after institution of the restricted reimbursement program.

### Optional special authorization policy

Alberta Health and Wellness (AHW) provides drug coverage for a defined formulary to all residents of Alberta Canada over age 65 through Alberta Blue Cross, the details of which can be found elsewhere (https://www.ab.bluecross.ca/ ). Prior to November 15, 2005, four quinolones (ciprofloxacin, levofloxacin, norfloxacin and ofloxacin) were a general benefit on the Alberta Blue Cross formulary (meaning that they would be reimbursed without restriction). On November 15, 2005, AHW established a new formulary restriction policy for quinolones (entitled “optional special authorization” (OSA) (Additional file 1)) where physicians could voluntarily enroll and become a designated quinolone prescriber, and at the same time, AHW added two new quinolones (gatifloxacin and moxifloxacin) to the formulary. To facilitate physicians’ choice of antimicrobials, a guide to prescribing restrictions for quinolones and a focused educational package was mailed to all physicians along with a “consent to participate” form. Patients of physicians who were “designated” prescribers (i.e. they had returned their consent form, agreeing to prescribe quinolones in accordance with the prescribing guidelines (Additional file 1)) had their quinolone prescription reimbursed without further paperwork, while for patients of physicians not enrolled in the program, these physicians had to complete and fax a paper-based prior authorization form to Alberta Blue Cross before the prescription would be reimbursed. Approval would generally be returned within 24 hours if the prescription was consistent with prescribing guidelines. This policy applied to three of the four quinolones which were reimbursed prior to November 2005 (ciprofloxacin, levofloxacin and ofloxacin – it did not apply to norfloxacin), as well as two new quinolones (gatifloxacin and moxifloxacin) which were added to the formulary November 15, 2005 (Additional file 1).

### Study population

Given that coverage of pharmaceuticals is provided to all Albertans ≥ age 65 (and thus complete data on prescribing is available only for this cohort), our study was restricted to Albertans aged ≥ 65. Patients were included in the cohort if they had an outpatient visit to a primary care physician for acute exacerbation of chronic bronchitis, community acquired pneumonia, upper respiratory tract infection or urinary tract infection, common out-patient infections for which quinolones may be indicated (See Additional file 2 for a list of International Classification of Disease 9^th^ version [ICD-9] diagnosis codes used to define the infectious conditions). The study period included the two year period preceding, and one year after implementation of the restricted reimbursement program (effective November 15 2005).

We defined a **unique index visit** as a physician claim for one of the infections of interest, with no similar claim for the same infection in the preceding 30 days. Outpatient physician claims during the 30 day period following each unique index visit were evaluated and if a claim for a different infection of interest was identified, then a new unique index visit was created.

Antibiotic use, as determined from the Alberta Blue Cross drug file of AHW, was assessed during the 30 day period following each unique index visit. The first antibiotic dispensed was considered to be associated with the infection preceding the prescription. If more than one antibiotic was dispensed during the 30 day follow-up period, and was dispensed on different days, then each antibiotic was counted as a separate antibiotic prescribing episode for the unique index visit preceding it.

While we were interested in primary care physicians who were enrolled in the OSA program, those who were not enrolled in the OSA program were still subject to new prescribing rules. As such, we examined the use of antibiotics among primary care physicians who were and were not enrolled in the OSA program. This administrative data cohort was used to assess the primary objective.

To achieve the secondary objective regarding appropriateness of quinolone prescribing, we undertook a chart review in primary care physician offices. A convenience sample of primary care physicians in Calgary, Alberta as well as rural surrounding areas were contacted and invited to participate. For physicians consenting to a chart review, we obtained a list of patients they had assessed in their clinic for at least 1 of the infections of interest (see ICD9 codes, Additional file 2) in the year before and/or the year after implementation of the restricted reimbursement program from AHW. A trained medical records analyst reviewed up to 20 randomly selected charts from each physician’s practice, for each of these two periods using structured and standardized data collection forms.

### Measures

The primary outcome measure (assessed using the administrative data cohort) was the use of a quinolone in the 30 day period following a unique index visit for a urinary tract infection, upper respiratory tract infection, acute exacerbation of chronic bronchitis and community acquired pneumonia. We also assessed the use of an antibiotic that was not restricted within the restricted reimbursement program in this 30 day period.

For patients noted on chart review to have a physician visit for one of the infections of interest, the secondary outcome measure included the proportion of visits that were associated with prescription for a fluroquinolone *consistent * with the restricted reimbursement prescribing criteria.

### Statistical analysis

*For the primary objective* (to measure the impact of a restricted reimbursement program for quinolones on the use of quinolones for patients with common outpatient infections) the unit of analysis was the unique index visit for any of the four common infections. In an initial exploratory analysis we compared the rate of quinolone antibiotic use, other antibiotic use and no antibiotic use for the infectious conditions of interest, for the pre and post OSA time periods. We further stratified this by prescriber designation (enrolled and not-enrolled in the OSA program), and by infection of interest.

The impact of the restricted reimbursement program on the outcome of quinolone use was examined using segmented linear regression analysis of interrupted time series data. Broadly speaking, the goal of this type of analysis is to examine for a level change (i.e. an abrupt change in quinolone prescribing) or a slope change (i.e. a change in the rate at which prescriptions of quinolones are changing) [15,16]. This analysis takes into account pre-OSA prescribing trends and potential autocorrelation or seasonal influences that may be present [15]. As an initial step in the analysis a generalized estimating equations (GEE) model was used to obtain an adjusted monthly rate of quinolone use. To calculate rates of quinolone use the dependent variable was prescription of a quinolone (yes/no), in the 30 day period following a unique index visit for each of the four infections. The monthly rates were adjusted for age, sex and whether the physician was enrolled in the OSA program. The GEE approach is an extension of standard logistic regression which adjusts for correlation among observations (i.e., the potential correlation and clustering of physician prescribing practices) [16]. Although other analytical methods, such as mixed-effects generalized linear models, would allow for more detailed modeling of effects that contribute to the variance and covariance of observations, we selected GEE as it provides ‘population-average’ effect that has familiar interpretation and is more useful when estimating effects of an intervention at a population level [17,18]. Adjusted monthly rates of quinolone use were included in the segmented linear regression models as the dependent variable. Segmented regression models generally fit a least squares regression line in each segment and assume a linear relationship between the independent variable and the outcome within each segment [19]. The most parsimonious segmented linear regression model was achieved using a backward elimination approach of non-significant variables from the full segmented regression. The Durbin-Watson statistic was calculated to test for a serial autocorrelation of the residuals.

For the secondary objective (examining the use of quinolones consistent with the restricted reimbursement prescribing criteria), we performed descriptive analyses using chi-square tests to assess differences between proportions for the pre and post program periods.

Ethics approval was obtained from the Conjoint Health Research Ethics Board in Calgary, and all physicians who participated in the chart review provided informed consent. The study funders had no role in study design or the collection, analysis, and interpretation of data or the writing of the article, nor any role in the decision to submit it for publication. The researchers acted independently from study funders, and the researchers had access to all of the data and performed all analyses independently.

## Results

### Administrative data cohort

#### Baseline characteristics

The study population consisted of 170,247 individuals who were seen at least once during the three year period for one of the infections of interest (Table 1). These individuals were seen a total of 436,888 times for these conditions of interest, resulting in a total of 397,534 unique index visits. Of these visits, 341,899 (86.0%) were to physicians who enrolled in the optional special authorization program.

**Table 1:** Characteristics of study population and unique index infection visits over the three year study period

**Overall Study Population (n = 170,247)**	
Median age (IQR) at beginning of OSA program	74 (69, 80)
Female (n,%)	97,568 (57.3%)
Death within 30 days post index visit for the overall population (n,%)	1,270 (0.8%)
Any antibiotic prescription within 30 days post index visit for the overall population (n,%)	86,436 (50.8%)
Unique Index Visits for Infections of Interest (n = 397,534)	
AECB	119,215 (30.0%)
Proportion prescribed any antibiotic within 30 days 35%	
URTI	185,946 (46.8%)
Proportion prescribed any antibiotic within 30 days 62%	
Pneumonia	37,869 (9.5%)
Proportion prescribed any antibiotic within 30 days 48%	
UTI	54,504 (13.7%)
Proportion prescribed any antibiotic within 30 days 71%	
Patients with a Unique Index Visit ^1^ (n = 397,534)	
Antibiotic Use Within 30 days Following Unique Index Visit ^1^	
No antibiotic use	200,794 (50.5%)
Antibiotics other than macrolides and quinolones	77,586 (39.4%)
Macrolides	61,331 (31.2%)
Quinolones	57,823 (29.4%)
*Levofloxacin*	*32,631 (56.4%)*
*Ciprofloxacin*	*22,411 (38.8%)*
*Moxifloxacin*^2^	*2,578 (4.5%)*
*Gatifloxacin*^2^	*151 (0.3%)*
*Ofloxacin*^2^	*52 (0.1%)*

^**1**^A unique index visit could result in antibiotics being dispensed on separate days during the 30 follow-up period, and as such, numbers do not add up to 397,534.

^**2**^only available on formulary as of Nov 15, 2005.

The rates and proportion of visits for each infection were stable over the three year period. The majority of unique index visits were for acute exacerbation of chronic bronchitis or for upper respiratory tract infection (76.8%) (Table 1). Antibiotics were prescribed within 30 days for 49.5% of the unique index visits (Table 1). Of the 196,740 unique index visits followed by an antibiotic prescription, additional antibiotics were prescribed on a subsequent day within the 30 day period for 20% of the unique index visits. For patients who received an antibiotic prescription, quinolone antibiotics were dispensed on 29.4% of occasions.

#### Overall Antibiotic and Quinolone antibiotic use for OSA and NonOSA physicians

**Figure 1:**
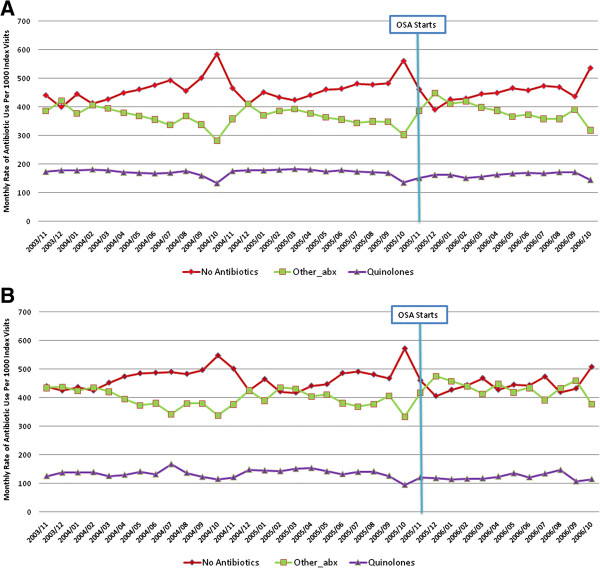
**Monthly rate of quinolone, other antibiotic, and no antibiotic use per 1000 index visits for all infections, for physicians enrolled (Panel A) and not enrolled (Panel B) in the optional special authorization program. **Panel **A**: Physicians enrolled in the optional special authorization program. Panel **B**: Physicians not enrolled in the optional special authorization program.

The rate of quinolone use, other antibiotic use and no antibiotic use post index visit for the infectious conditions of interest was similar for physicians enrolled and those not enrolled in the optional special authorization program (Figure 1: Panel A (physicians enrolled in the optional special authorization program) and Panel B (physicians not enrolled in the optional special authorization program). Given this and the fact that both groups of physicians were subject to new prescribing rules for quinolones, analyses below include patients seen by both physician groups. Of note, Figure 1 displays a seasonal drop in the use of antibiotics in October of each year – this corresponds to physician claims for influenza which we postulate relates to visits for influenza vaccination.

#### Overall antibiotic and quinolone antibiotic use before and after implementation of the OSA program

For unique index visits, there was a small increase in the probability of receiving an antibiotic prescription after implementation of the OSA program (53.7% in the two years before implementation of OSA, and 54.8% in the year after implementation (p < 0.0001). Although statistically significant, this change is small.

**Figure 2:**
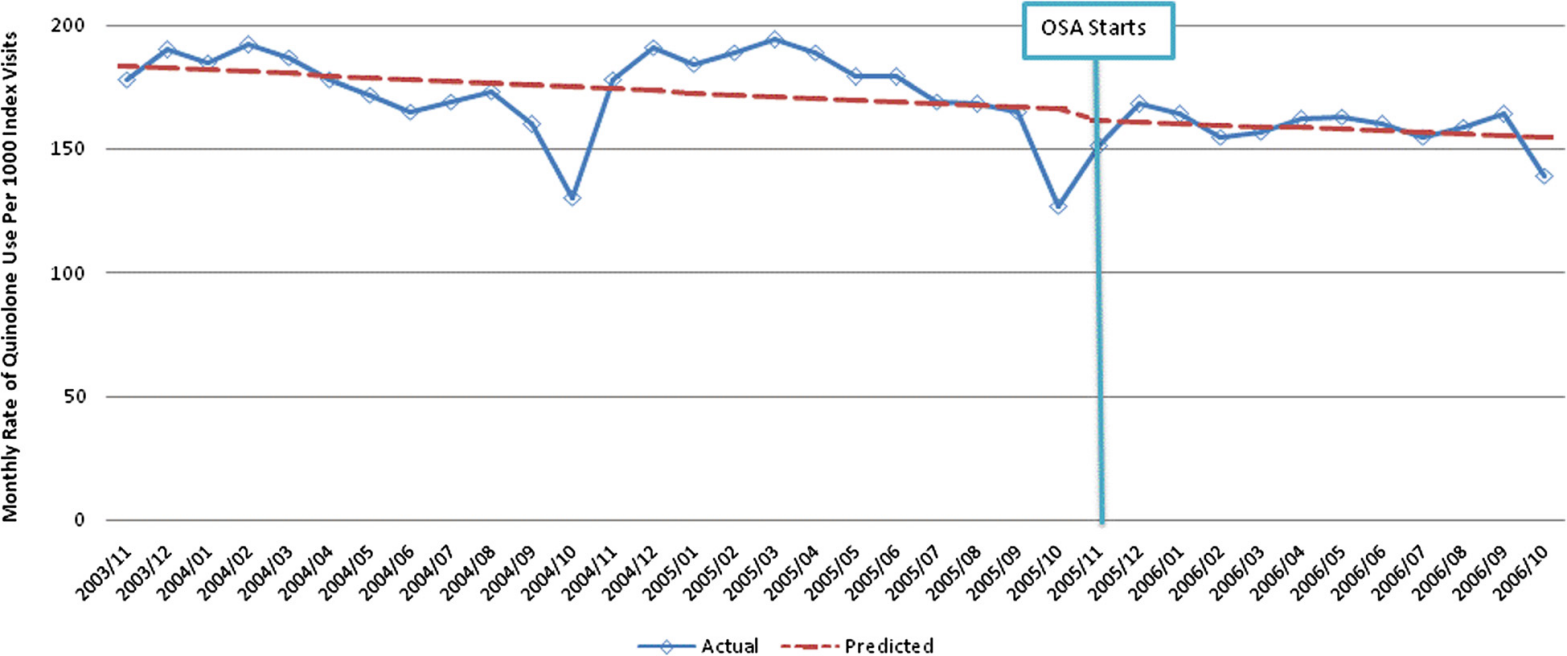
Time series of monthly adjusted* rates of quinolone use per 1000 index visits for all infections of interest. Fitted trend lines show predicted values from the segmented regression model.

Using segmented regression models adjusting for age, sex and physician enrollment in the OSA program, there was no statistically significant change in the rate of quinolone use (level change −3.5 (95% CI −5.5, 1.4) prescriptions per 1000 index visits; p = 0.74)), and there was no change in the slope of quinolone use (p = 0.95), following implementation of the OSA program (Figure 2). Of note, there was no significant level or slope change in the rate of quinolone use after implementation of the OSA program for pneumonia or AECB, after controlling for confounders. There was, however, a significant level change in the rate of quinolone use for UTI (−33.6 (95% CI: -23.8, -43.4) prescriptions (p < 0.001) and URTI (−16.1 (95%CI: -11.6, -20.6) prescriptions (p < 0.001) per 1000 unique index visits, but no significant change in the slope.

In addition to implementing the OSA program on Nov 15, 2005, two new quinolone antibiotics (both brand name products) were also added to the Alberta Blue Cross formulary on the same date. Therefore it may be expected that quinolone use would increase (irrespective of the OSA program), and that this might influence the trends in quinolone use over time. To examine the implications of the simultaneous addition of these new agents, we repeated analyses before and after implementation of the OSA program using alternate definitions of quinolone antibiotics – specifically considering the impact on the use of levofloxacin for respiratory diagnoses and ciprofloxacin for urinary tract infection.

**Figure 3:**
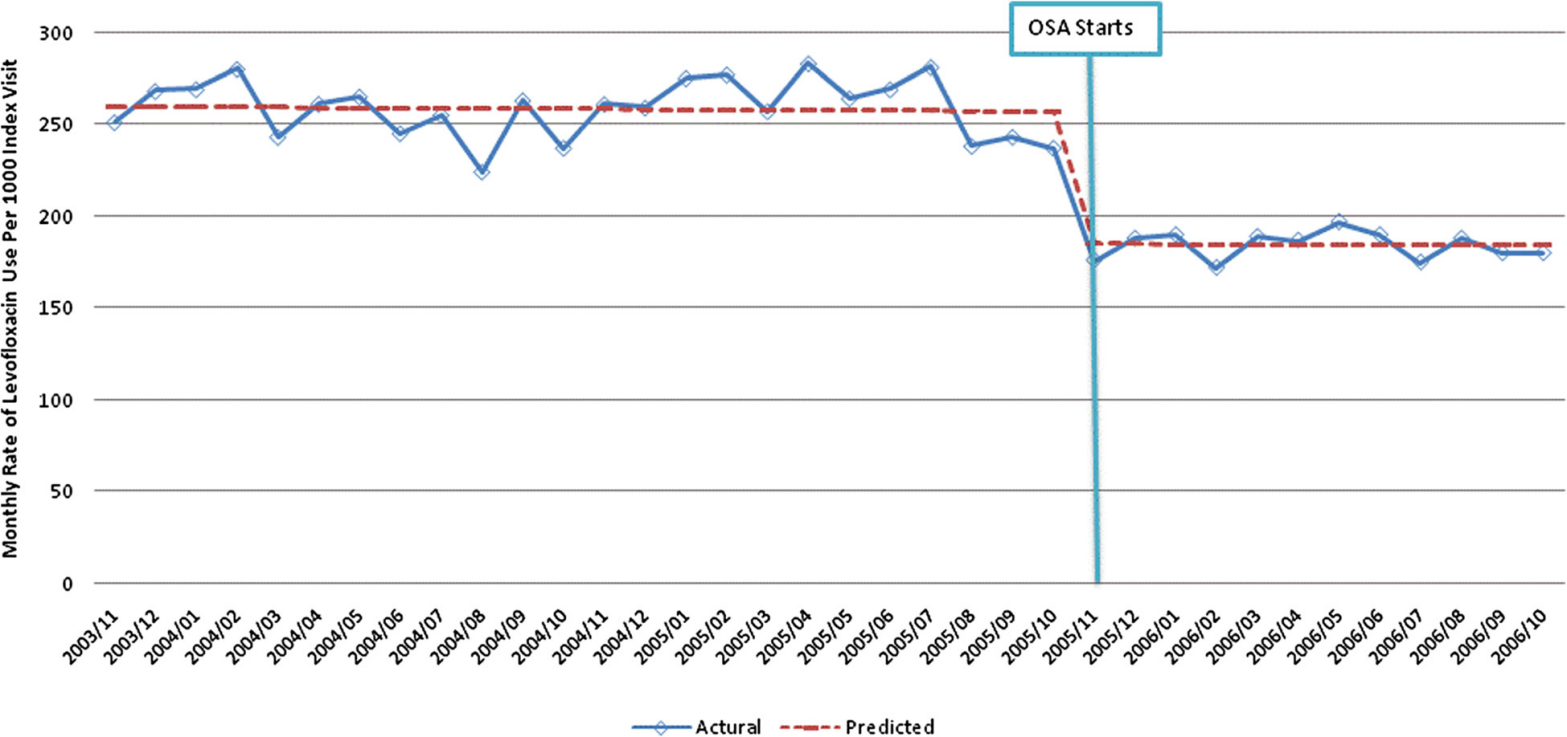
Time series of monthly adjusted* rates of levofloxacin use per 1000 index visits for acute exacerbation of chronic bronchitis. Fitted trend lines show predicted values from the segmented regression model.

Given that ciprofloxacin is the predominant quinolone used for urinary tract infections, we examined the use of ciprofloxacin after implementation of the OSA program. Among antibiotic users, there was a level change in the rate of ciprofloxacin use for urinary tract infections of −69.1 (95%CI: -49.5, -88.7) prescriptions per 1000 unique index visits after implementation of the OSA program (p < 0.001)). Similarly, as levofloxacin is the predominant quinolone used for AECB, URTI and pneumonia, we examined the use of levofloxacin before and after implementation of the OSA program. Among antibiotic users, there was a level change in the rate of levofloxacin use of −74.2 (95%CI: -64.4, -83.8) prescriptions per 1000 index visits for AECB (Figure 3), -62.9 (95%CI: -57.8, -68.0) prescriptions per 1000 index visits for URTI and −99.1 (95%CI: -78.3, -119.9) prescriptions for pneumonia after implementation of the OSA program (p < 0.001), with no significant change in the slope of prescribing (p = 0.95). Of note, the use of the brand-name quinolones that were not funded prior to the OSA program but were funded as of the date of OSA initiation (gatifloxacin and moxifloxacin) increased after implementation of the OSA program (data not shown), explaining the smaller reduction in use of quinolones overall.

#### Impact on mortality, hospitalization, and subsequent primary care visits within 30 days

For patients presenting with any of the four infections of interest used to define unique index visits, the thirty day mortality risk was stable before (0.3%) and after (0.3%) implementation of the optional special authorization program (p = 0.54). After implementation of the OSA program, there was a very small increase in the proportion of patients requiring hospitalization (all cause) within 30 days of an outpatient infection claim (4.9% vs 5.2%, p = 0.0001), though there were no differences in the proportion of patients admitted for one of the four infections of interest (1.4% vs 1.4%, p = 0.2). The proportion of patients with any of the four infections used to define unique index visits who had a subsequent outpatient physician claim in the next 30 days increased from 55.6% to 56.5% (p < 0.001) after implementation of the optional special authorization program, though this change is unlikely to be of clinical significance.

### Chart review cohort

#### Baseline characteristics of patients

Chart reviews were conducted in a convenience sample of 60 consenting physician practices (127 primary care physicians were approached), corresponding to 3846 patient visits. On average, 64 patient visits were reviewed in each primary care physician’s office. However, upon review of the chart, it was determined that only 1681 (43.7%) of patient visits with a billing claim for an infection of interest in fact had one of the four infections of interest. A total of 804 (47.8%) of patient visits were before and 877 (52.7%) were after implementation of the OSA program. On average, 15 patient visits with infections of interest per primary care physician were reviewed before, and 15 patient visits were reviewed after implementation of the OSA program (Table 2).

**Table 2:** Baseline characteristics of chart review cohort

**Chart Review Information**	
Total number of charts reviewed	n = 3,846
Number of GP practices visited	n = 60
Study Population - Patient visits confirmed to have an infection of interest (%)	1,681 (43.7%)
Median age (IQR) for patients with visits, years	73 (69, 79)
Female, n (%)	1,110 (66.0%)
Number of infection visits before OSA program implementation, n (%)	804 (47.8%)
Number of infection visits after OSA program implementation, n (%)	877 (52.3%)
Infections of interest among included patient visits	n = 1,681
AECB, n (%)	101 (6.0%)
URTI, n (%)	1,070 (63.6%)
Pneumonia, n (%)	146 (8.7%)
UTI, n (%)	366 (21.9%)
Antibiotic Use Following Infection of Interest	n = 1,681
No Antibiotic Use	536 (31.9%)
Any Antibiotic Use	1,145 (68.1%)
*Quinolone Use*	*351 (30.7%)*

#### Overall Antibiotic and quinolone antibiotic use before and after implementation of the OSA program

Overall, there was no change in the proportion of visits for infection which were associated with an antibiotic prescription (p = 0.86) after OSA implementation, for both OSA enrolled and non-enrolled physicians. Among visits for infection treated with an antibiotic, there was no change in quinolone antibiotic use after implementation of OSA program. Among quinolone prescriptions, 42.5% and 58.5% were consistent with formulary guidelines before and after the implementation of OSA, respectively (p = 0.002). This change was stable over time, with 58.6% of quinolone prescriptions being guideline compliant in the last six months of the year after OSA implementation, and seasonal variation in appropriateness of prescribing was small.

## Discussion

In this large community-based cohort, we found no change in prescription of the overall quinolone class of antibiotics for four common infections after implementation of a new restricted reimbursement policy for quinolones. However, stabilization in the use of quinolones could be construed as encouraging, given that two new quinolones were added to the formulary at the same time as the restricted reimbursement policy was implemented. Moreover, we observed a significant reduction in the use of levofloxacin for AECB and URTI, and in the use of ciprofloxacin for urinary tract infection, conditions in which quinolones are frequently overused. While the lack of difference observed between OSA and non-OSA enrolled physicians might mean that the program had minimal impact on prescribing, primary care physicians who were not enrolled in the OSA program were also subject to greater quinolone prescribing restrictions since they were required to apply for traditional prior authorization before prescribing quinolones. It is possible that any changes observed were due to the receipt of educational materials on the appropriate use of quinolones, since both groups of physicians received educational materials.

As noted, this analysis was confounded by the addition of two new quinolones to the Alberta Blue Cross formulary in conjunction with the OSA program. When we considered only quinolones that were on the formulary prior to the OSA program, quinolone use decreased by 6-10% for the different infections. The results of our chart review were consistent with those observed in our cohort derived from administrative data. In addition, the chart review demonstrated a >15% increase in the proportion of quinolone prescribing that was consistent with formulary guidelines. Finally, the implementation of this program did not appear to have a significant adverse impact on hospitalization, death, or use of physician services in the subsequent 30 days.

The new restricted reimbursement policy may have been more effective at guiding physician prescribing if it included repeated physician education or ongoing feedback to physicians on their antimicrobial prescribing [4], since several studies suggest that repeated education, with or without academic detailing [20-22], may be more effective at changing physician behaviour. A 2005 Cochrane review examining interventions to improve outpatient antibiotic prescribing suggests that multi-faceted interventions combining physician, patient and public education in a variety of venues and formats is most successful in reducing antibiotic prescribing for inappropriate indications [23].

A limitation of our study, which was confirmed in our chart review, is that the diagnostic information obtained from physician claims was often inaccurate. However, these codes were intentionally selected to define a broad cohort of patients who *might* receive antibiotic therapy and it is unlikely that potential misclassification would differ before or after institution of the restricted reimbursement program, or by physician enrollment status. Another limitation is the potential for selection bias in that the primary care physicians who consented to participate in the chart review portion of the study might be different than the broader primary care physician population. It is reassuring that the observed lack of change in quinolone prescribing was consistent in both the chart review and the administrative cohort and physicians were unaware that their prescribing would be audited in the year following OSA implementation. Our analysis is limited by its observational design, and changes observed might be due to factors other than the optional special authorization program. However, a previous study examining the impact of a change in drug policy found similar results in both a cluster randomized trial and a simultaneously conducted observational analysis [4] which (like our study) controlled for potential confounders and temporal trends.

Finally, our study assessed compliance with formulary restrictions, and not necessarily what might be construed as “best” practice. However, all data related to compliance with quinolone antibiotic prescribing restrictions from the patient charts were reviewed by a physician who was unaware of whether the patient was seen before or after implementation of the OSA program. This review was undertaken to determine whether such use might be deemed appropriate clinically, based on results of resistance testing or a clinical history of severe pneumonia. Only 4.3% of cases where quinolone prescriptions were not formulary guideline compliant were felt to be appropriate clinically (as defined above), with no difference before or after OSA implementation.

## Conclusion

We found that a new restricted reimbursement policy was associated with stabilization in the use of quinolones in patients with common outpatient infections despite the inclusion of two new quinolone antibiotics on the provincial formulary, and was associated with an improvement in the appropriateness of quinolone antibiotic prescribing in the short-term. Future prescribing initiatives should assess whether ongoing provision of educational materials over time can enhance prescribing in accordance with formulary guidelines.

## Competing interests

All of the authors declare that they have no relevant competing interests.

## Authors’ contributions

Dr M, L, T, and H made substantial contributions to study conception and design, drafting and critical revision. Dr M, H and G have all been involved with data analysis, and interpretation of data, and Dr G drafted the methods and results sections. All authors have approved the final version.

## Pre-publication history

The pre-publication history for this paper can be accessed here:

http://www.biomedcentral.com/1472-6963/12/290/prepub

## Supplementary Material

Additional file 1 Blue cross. Click here for file

Additional file 2 ICD-9 Diagnosis Codes for Outpatient Infections of Interest. Click here for file
