# Supplementary material for: Evaluating the impact of a novel restricted reimbursement policy for quinolone antibiotics: A time series analysis
Source: BMC Health Serv Res. 2012 Aug 30;12:290. doi: 10.1186/1472-6963-12-290 (PMC3470979; doi:10.1186/1472-6963-12-290)
Supplement: Additional file 1 — Blue cross. [file 1472-6963-12-290-S1.doc]

**Appendix 1:**
