# Supplementary material for: Evaluating the impact of a novel restricted reimbursement policy for quinolone antibiotics: A time series analysis
Source: BMC Health Serv Res. 2012 Aug 30;12:290. doi: 10.1186/1472-6963-12-290 (PMC3470979; doi:10.1186/1472-6963-12-290)
Supplement: Additional file 2 — ICD-9 Diagnosis Codes for Outpatient Infections of Interest. [file 1472-6963-12-290-S2.doc]

**Appendix 2: ICD-9** Diagnosis Codes for Outpatient Infections of Interest

| **CONDITION** | **ICD-9 Diagnosis Codes** |
| --- | --- |
| **Urinary tract infection** |  |
|  | 595 Cystitis |
|  | 595.0 Acute cystitis |
|  | 595.1 Chronic interstitial cystitis |
|  | 595.2 Other chronic cystitis |
|  | 595.3 Trigonitis |
|  | 595.4 Cystitis in diseases classified elsewhere |
|  | 595.8 Other specified types of cystitis |
|  | 595.9 Cystitis unspecified |
| **Upper respiratory tract infection** |  |
|  | 460 Acute nasopharyngitis |
|  | 462 Acute pharyngitis |
|  | 463 Acute tonsillitis |
|  | 464 Acute laryngitis and tracheitis |
|  | 464.0 Acute laryngitis |
|  | 464.1 Acute tracheitis |
|  | 464.2 Acute laryngotracheitis |
|  | 464.3 Acute epiglottitis |
|  | 464.4 Croup |
|  | 465 Acute upper respiratory infection of multiple sites |
|  | 465.0 Acute laryngopharyngitis |
|  | 465.8 Other multiple sites |
|  | 465.9 Unspecified sites |
|  | 466 Acute bronchitis and bronchiolitis |
|  | 466.0 Acute bronchitis |
|  | 466.1 Acute bronchiolitis |
| **Acute exacerbation of chronic bronchitis** |  |
|  | 490 Bronchitis, not specified as acute or chronic |
|  | 491 Chronic bronchitis |
|  | 491.0 Simple chronic bronchitis |
|  | 491.1 Mucopurulent chronic bronchitis |
|  | 491.2 Obstructive chronic bronchitis |
|  | 491.8 Other chronic bronchitis |
|  | 491.9 Unspecified chronic bronchitis |
|  | 492 Emphysema |
|  | 492.0 Emphysematous bleb |
|  | 492.8 Other emphysema |
|  | 493 Asthma |
|  | 493.0 Extrinsic asthma |
|  | 493.1 Intrinsic asthma |
|  | 493.2 Chronic obstructive asthma |
|  | 493.9 Asthma, unspecified |
|  | 494 Bronchiectasis |
|  | 495 Extrinsic allergic alveolitis |
|  | (495.0 – 495.9) |
|  | 496 Chronic airway obstruction, not elsewhere classified |
| **Pneumonia** |  |
|  | 480 Viral pneumonia |
|  | 480.0 Due to adenovirus |
|  | 480.1 Due to RSV |
|  | 480.2 Due to parainfluenza virus |
|  | 480.8 Due to other virus not elsewhere classified |
|  | 480.9 Unspecified |
|  | 481 Pneumococcal pneumonia |
|  | 482 Other bacterial pneumonia |
|  | 482.0 Due to Klebsiella pneumoniae |
|  | 482.1 Due to Pseudomonas |
|  | 482.2 Due to Hemophilus influenzae |
|  | 482.3 Due to Streptococcus |
|  | 482.4 Due to Staphylococcus |
|  | 482.8 Due to other specified bacteria |
|  | 482.9 Due to other specified organism |
|  | 483 Pneumonia due to other specified organisms |
|  | 483.0 Mycoplasma pneumonia |
|  | 483.1 Chlamydia |
|  | 483.8 Other specified organism |
|  | 484 Pneumonia in inf. diseases classified elsewhere |
|  | 484.1 Penumonia in cytomegalic inclusion disease |
|  | 484.3 Pneumonia in whooping cough |
|  | 484.5 Pneumonia in anthrax |
|  | 484.6 Pneumonia in aspergillosis |
|  | 484.7 Pneumonia in other systemic mycoses |
|  | 484.8 Pneumonia in other inf disease class elsewhere |
|  | 485 Bronchopneumonia, organism unspecified |
|  | 486 Pneumonia, organism unspecified |
|  | 487 Influenza |
|  | 487.0 With pneumonia |
|  | 487.1 With other respiratory manifestations |
|  | 487.8 With other manifestations |
